# Unequal Burdens: Exploring Racial Differences in Sarcoidosis-Related Deaths Due to Pulmonary Hypertension Versus Fibrosis in the United States (1999-2023)

**DOI:** 10.7759/cureus.103785

**Published:** 2026-02-17

**Authors:** Muhammed Umer, Muhammad Faizan, Fizzah Mohammad Hanif, Salim Surani

**Affiliations:** 1 Internal Medicine, Saint Michael's Medical Center, Harrison, USA; 2 Medicine, Dow University of Health Sciences, Karachi, PAK; 3 Internal Medicine, Sir Syed Medical College, Karachi, PAK; 4 Medicine, Valley Coastal Bend VA, Harlingen, USA; 5 Medicine, University of Houston, Houston, USA; 6 Medicine, Aga Khan University, Nairobi, KEN; 7 Anesthesiology, Mayo Clinic, Rochester, USA

**Keywords:** mortality, pulmonary fibrosis, pulmonary hypertension, racial differences, sarcoidosis

## Abstract

Introduction

Sarcoidosis disproportionately affects Black Americans, who report a higher incidence (17.8 per 100,000) compared to White Americans (8.1 per 100,000) and also experience greater disease severity, leading to an overall worse prognosis. Despite these trends, racial differences in cause-specific mortality among patients with sarcoidosis remain unexplored. In light of this, we aimed to assess longitudinal trends in mortality rates attributed to pulmonary causes of death among individuals with comorbid sarcoidosis.

Methods

We conducted a retrospective repeated cross-sectional study to determine the cause of death attributed to pulmonary deaths with comorbid sarcoidosis in the United States from 1999 to 2023. The data were obtained from the Centers for Disease Control and Prevention’s Wide Ranging Online Data for Epidemiologic Research (CDC WONDER) database, which includes the underlying and contributing causes of death from all death certificates in the United States.

Results

Of the 24,156 pulmonary deaths with comorbid sarcoidosis identified, 61.1% were women, and nearly 50% were aged 55-74 years. The age-adjusted mortality rate (AAMR) increased from 2.7 (95% confidence interval (CI): 2.5-3.0) per million in 1999 to 4.8 (95% CI: 4.5-5.1) in 2023. The annual percentage change (APC) was +1.1% (95% CI: 0.85-1.35) from 2001 to 2018, with a marked rise from 2018 to 2020 (APC: +10.36%, 95% CI: 4.50-16.54). Pulmonary fibrosis (PF) and pulmonary hypertension (PH) constituted 70% of pulmonary deaths with sarcoidosis. When stratified by race, Black individuals are more likely to die from PH (37.3%), with a greater burden in Black women (40.5%). In contrast, White individuals most commonly died from pulmonary fibrosis (PF) (52.8%).

Conclusions

Pulmonary deaths in sarcoidosis have increased over the past two decades, with racial disparities evident in underlying phenotypes. Black individuals, especially Black women, are more likely to die from PH, while White individuals disproportionately die from fibrosis. These findings highlight the importance of phenotype-specific screening strategies and equity-focused clinical interventions.

## Introduction

Sarcoidosis is a multisystem disease characterized by the formation of noncaseating granulomas in various organs, most commonly the lungs. In the United States, the prevalence of sarcoidosis is estimated to be approximately 35.22 per 100,000 individuals [1]. Sarcoidosis disproportionately affects Black Americans, who report a higher incidence (17.8 per 100,000) compared to White Americans (8.1 per 100,000) and also experience greater disease severity, leading to an overall worse prognosis [2].

Pulmonary involvement is reported in over 90% of sarcoidosis cases, where granulomatous inflammation leads to fibrosis and damage to the lung parenchyma and airways [3]. Beyond pulmonary complications, sarcoidosis can also affect other vital organs, including the cardiovascular system. A previous study investigating trends and disparities in cardiovascular deaths among sarcoidosis patients highlighted substantial racial differences, with Black individuals exhibiting higher cardiovascular mortality rates compared to White individuals [2]. However, significant uncertainty persists regarding the comparative mortality burden of pulmonary hypertension (PH) and pulmonary fibrosis (PF) within the sarcoidosis population. Identifying these patterns is essential, as they could improve management strategies for this high-risk population.

Despite the clinical relevance of these trends, real-world data on sarcoidosis mortality from pulmonary complications are limited. Therefore, we aimed to assess the longitudinal trends in mortality rates attributed to pulmonary causes of death with comorbid sarcoidosis in the United States.

## Materials and methods

Data source

We conducted a retrospective repeated cross-sectional study to evaluate longitudinal trends in mortality rates attributed to pulmonary deaths with comorbid sarcoidosis in the United States from 1999 to 2020. The data were obtained from the Centers for Disease Control and Prevention’s Wide Ranging Online Data for Epidemiologic Research (CDC WONDER) database, which includes the underlying and contributing causes of death from all death certificates in the United States. The International Classification of Diseases, Tenth Revision (ICD-10) is used to classify the causes of death for the years 1999 to 2020.

Using the CDC WONDER database, diseases of the respiratory system (J00-J99, excluding J40 to J4a) and PH-related deaths (I27.0, I27.2, I27.8, and I27.9) were listed as the underlying causes of death, and sarcoidosis (D86) was listed as the contributing cause of death (Appendices). This ensured that deaths were primarily attributed to a specific pulmonary process (e.g., PF or PH), with sarcoidosis acting as the systemic comorbidity influencing the disease course. The World Health Organization defines the underlying cause of death as the disease or injury that initiates a sequence of events that leads directly to death [4]. Individuals with unknown causes of death stated on the death certificates at the time of death were excluded. Those aged 24 years and under were excluded due to data confidentiality constraints, as deaths were considered subnational in this age group. Furthermore, pulmonary sarcoidosis-related deaths are exceedingly rare in this age group. Moreover, pulmonary sarcoidosis-related deaths are exceedingly rare in this younger population and do not represent the typical epidemiologic or clinical phenotype. Including such sparse data could introduce disproportionate variance and distort trend estimation in age-adjusted mortality analyses.

**Figure 1:**
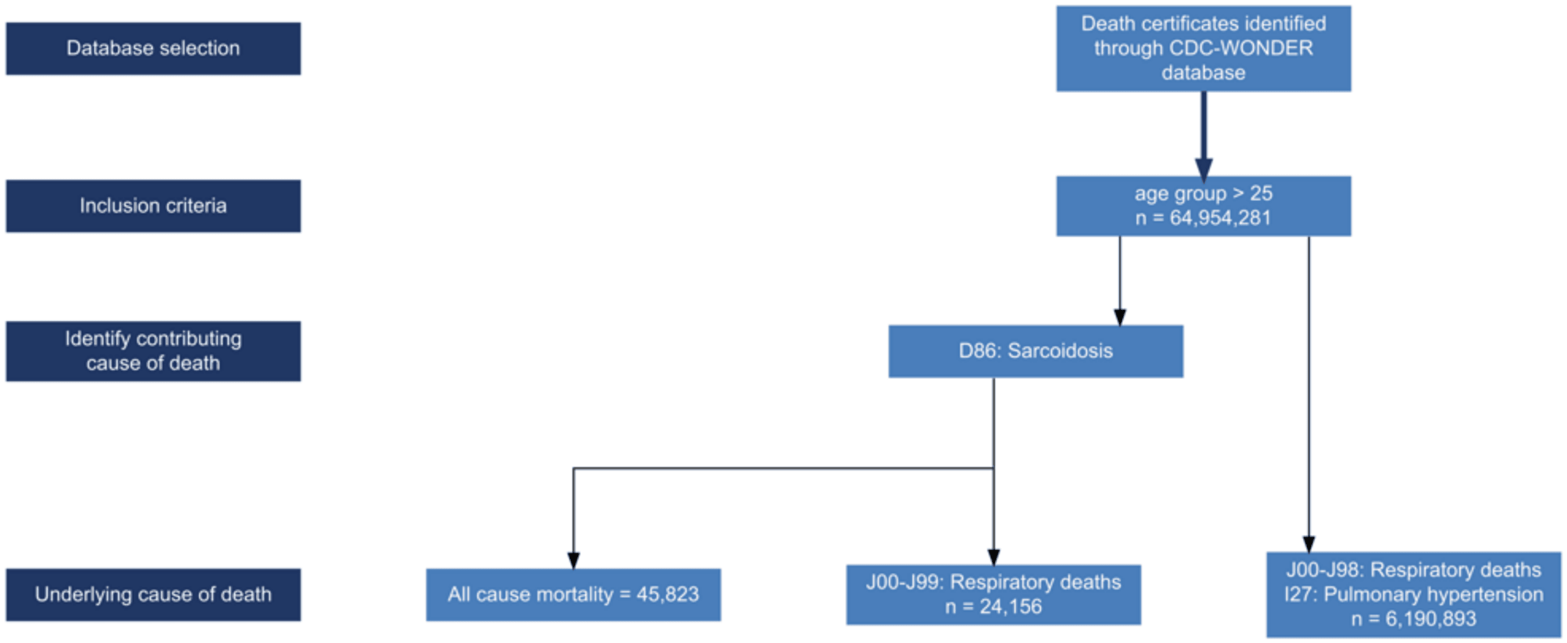
Flowchart depicting patient selection and study design CDC WONDER: Centers for Disease Control and Prevention’s Wide Ranging Online Data for Epidemiologic Research

The patient selection process is depicted in Figure 1. This methodology has been validated in another similar research [5]. Institutional review board approval and informed consent were not required, as the data were deidentified and are publicly available.

Statistical analysis

Age-adjusted mortality rate (AAMR) was used to quantify and compare mortality rates. Age-adjustment is a statistical process applied to rates of disease, death, injuries, or other health outcomes that allows communities with different age structures to be compared. AAMR was calculated per 1,000,000 population, adjusted to the 2000 U.S. Standard Population using the direct method. The denominator of 1 million was selected to provide clarity in reporting trends, as 100,000 produced AAMRs below 1.0, which obscured variability. Annual percentage change (APC) was calculated to evaluate trends in mortality over time. 

Joinpoint regression analysis (National Cancer Institute’s Joinpoint Trend Analysis Software, Version 5.3) was used to identify significant changes in trend and calculate APCs with 95% confidence intervals (CIs). A maximum of three joinpoints and the Monte Carlo permutation method with a two-sided alpha of 0.05 were used for statistical analysis. All rates were stratified by race (Black and White), sex, and underlying pulmonary cause of death. Furthermore, tests of parallelism and coincidence were conducted to compare mortality trends across population groups. The test of parallelism determined whether trends were similar across groups despite differences in absolute mortality rates, while the test of coincidence ruled out statistically identical trends.

To analyze mortality disparities, we calculated mortality rate ratios (RR) with 95% CIs to compare mortality from pulmonary complications within and across demographic groups. We used total death counts from 1999 to 2023 and total population-years to derive crude rates for our cohorts. The rate ratio is the ratio of mortality rates between the two groups or within the same group (e.g., Black mortality rate PH/White mortality rate PH) or (Black mortality PH/Black mortality PF). Crude rates were used because AAMR cannot be calculated due to small annual death counts. 

While CDC WONDER provides data for other racial and ethnic categories (e.g., Asian, Hispanic), these groups were not included in trend analyses due to low annual death counts. Specifically, many years had suppressed or sparse data (<10 deaths), making stratified Joinpoint regression unreliable. Therefore, we restricted stratified APC analyses to Black and White racial groups, for which mortality counts were sufficient for statistical analysis and interpretation.

## Results

**Figure 2:**
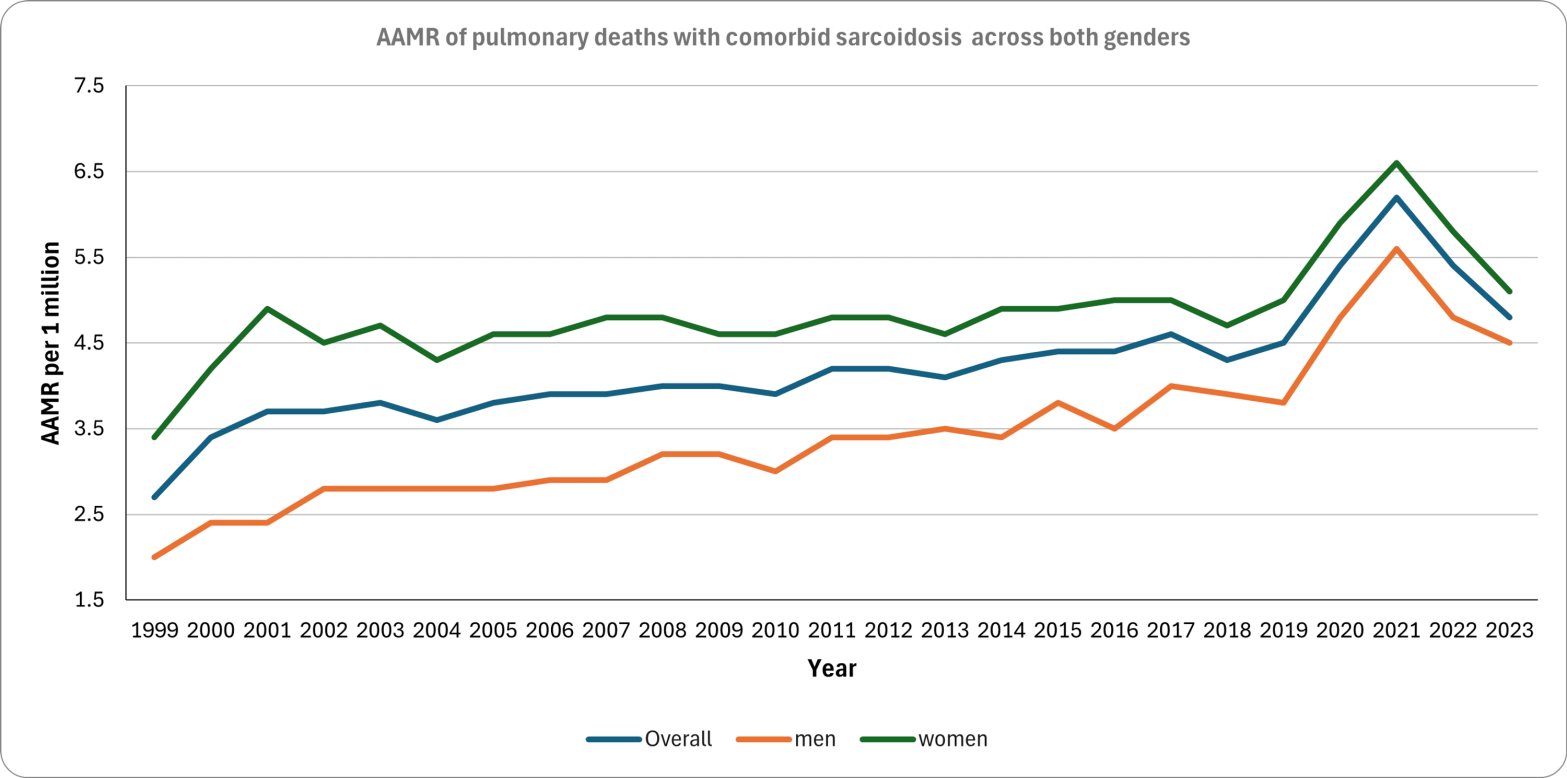
Trends in AAMR of respiratory failure with comorbid sarcoidosis between 1999 and 2023 stratified by sex AAMR: age-adjusted mortality rate

During the 24-year study period (1999-2023), 24,156 respiratory deaths with comorbid sarcoidosis were reported, accounting for 52.7% of all-cause deaths with comorbid sarcoidosis. Table 1 shows the baseline demographics and AAMR of patients who met the inclusion criteria. Women accounted for a higher proportion of sarcoidosis-related deaths overall (59.0% vs. 40.9%) and were overrepresented in pulmonary deaths with sarcoidosis comorbidity (61.1% vs. 38.9%), exhibiting a higher AAMR compared to men (4.80 vs. 3.22 per one million). About 50% of deaths occurred in the age group of 55-74 years in pulmonary deaths with sarcoidosis comorbidity. A prominent difference was seen in the AAMR of Black and White patients. The AAMR in Black patients was 19.3 (95% CI: 18.9-19.7), compared to 2.3 (95% CI: 2.2-2.4) in White patients. Overall, AAMR per one million individuals increased from 2.7 (95% CI: 2.5-3.0) in 1999 to 4.8 (95% CI: 4.5-5.1) in 2023 (Figure 2). Trend analysis revealed four distinct and statistically significant periods of change: an increase with an APC of 14.16 (95% CI: 4.42-24.8) from 1999 to 2001, followed by a stable but significant upward trend of 1.1 (95% CI: 0.85-1.35) from 2001 to 2018. A second sharp spike was observed from 2018-2020, with an APC of 10.36 (95% CI: 4.50-16.54), followed by a decline of -9.94 (95% CI: -14.56 to -5.08) from 2021 to 2023.

**Table 1:** Demographic characteristics of all-cause sarcoidosis and overall pulmonary mortality with age-adjusted rates for comorbid pulmonary-sarcoidosis deaths AAMR per 1,000,000 population (standardized to the 2000 U.S. Standard Population) was calculated specifically for the comorbid pulmonary-sarcoidosis cohort. For all-cause sarcoidosis and overall pulmonary deaths, demographic distributions are presented as raw counts (N). APCs and 95% CIs were calculated using Joinpoint Trend Analysis Software (National Cancer Institute), with statistical significance determined via the Monte Carlo permutation method (two-sided alpha = 0.05$) AAMR: age-adjusted mortality rate; APC: annual percentage change; CI: confidence interval

Demographic	All-cause death from sarcoidosis	Overall pulmonary deaths	Pulmonary causes of death in comorbid sarcoidosis	AAMR per 1 million for pulmonary causes of death with comorbid sarcoidosis X [95% CI]
	N = 45,823	N = 6,190,893	N = 24,156	4.21 [3.93 – 4.49]
Sex				
Men	N = 18,748 (40.9%)	N = 2,993,799 (48.4%)	8641 (38.9%)	3.22 [3.15 – 3.29]
Women	N = 27,075 (59.0%)	N = 3,197,094 (51.6%)	13,586 (61.1%)	4.80 [4.70 – 4.90]
Age at death, years, n (%)				
25-34	1009 (2.2%)	26,579 (0.4%)	264 (1.1%)	-
35-44	3813 (8.3%)	61,521 (1.0%)	1463 (6.1%)	-
45-54	8095 (17.7%)	197,914 (3.2%)	3895 (16.1%)	-
55-64	11,200 (24.4%)	597,795 (9.7%)	5917 (24.7%)	-
65-74	10,885 (23.8%)	1,284,216 (20.7%)	6292 (26.1%)	-
75-84	7961 (17.4%)	2,028,144 (32.8%)	4596 (19.0%)	-
85+	3040 (6.6%)	1,994,724 (32.2%)	1675 (6.9%)	-
Race, n (%)				
Native American	168 (0.3%)	33,866 (0.5%)	89 (0.%)	
Asian or Pacific Islander	348 (0.7%)	109,743 (1.7%)	165 (0.7%)	
Black	22,905 (48.6 %)	488,986 (7.6%)	10,806 (47.5%)	19.3 [18.9 – 19.7]
White	22,353 (47.4%)	5,554,171 (86.1%)	111,36 (48.9%)	2.3 [2.2 – 2.4]
Hispanic/Latino	1347 (2.9%)	266,591 (4.1%)	562 (2.5%)	
Census region, n (%)				
Northeast	9,091 (19.9%)	1,122,153 (18.1%)	4483 (20.2%)	4.60 [4.45 – 4.75]
Midwest	10,334 (22.6%)	1,468,431 (23.7%)	4880 (22.0 %)	4.20 [4.07 – 4.33]
South	19,780 (43.2%)	2,386,196 (38.5%)	9597 (43.2%)	5.00 [4.90 – 5.10]
West	6618 (14.4%)	1,214,113 (19.6%)	3267 (14.7%)	2.75 [2.65 – 2.85]

Cause of death stratified by race and gender in comorbid sarcoidosis

**Figure 3:**
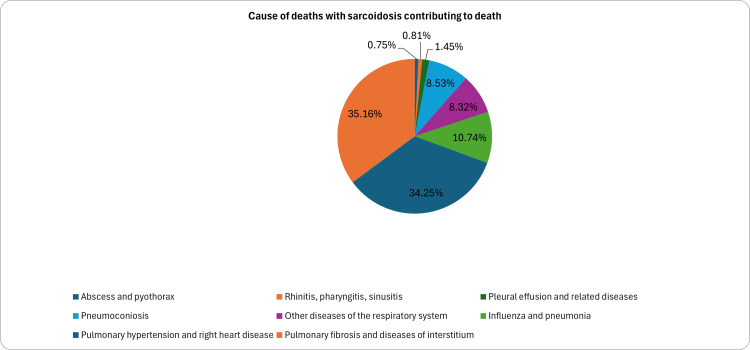
Cause of pulmonary deaths with comorbid sarcoidosis contributing to death

Figure 3 demonstrates the causes of pulmonary disease reported in sarcoidosis after excluding deaths from chronic obstructive pulmonary disease. Overall, PF and PH accounted for 70% of deaths. When stratified by race, it was observed that PH was the most common cause of death in 37.3% of the Black population, versus PF being the cause of death in 31.1% of the same population. PH, as the more common cause of death, was augmented by the rate ratio of 1.35 (95% CI: 1.14-1.59), which meant that Black patients were 35% more likely to die from PH than PF. An opposite trend was seen in the White population, where PF was the leading cause of death in around half the cases, with comorbid sarcoidosis, followed by 20.3% of deaths from PH (RR PH vs. PF: 0.45%; 95% CI: 0.38-0.53). This disparity was most prominent when comparing specific complications between cohorts. For instance, Black patients had a 12 times higher risk of dying from PH complications as compared to White patients (RR Black PH vs. White PH: 11.92; 95% CI: 9.96-14.28). Among the races, Black patients were 4x more likely to die from PF as compared to White patients (RR: 3.94, 95% CI: 3.36-4.62). These comparative rate ratios are summarized in Table 2.

**Table 2:** Comparative mortality RRs for PH and PF by racial cohort RRs were calculated by comparing the mortality rates of the respective cohorts. For inter-racial comparisons, White patients served as the reference group. For intra-racial comparisons, PF-related mortality served as the reference group. An RR >1.00 indicates a higher rate of mortality in the primary group, while an RR <1.00 indicates a lower rate. CIs were calculated based on the standard error of the log-transformed rates RR: rate ratio; PH: pulmonary hypertension; PF: pulmonary fibrosis; CI: confidence interval

Comparison group	Outcome metric	RR with 95% CI
Black vs. White	PH-related mortality	11.92 [9.96 - 14.28]
Black vs. White	PF-related mortality	3.94 [3.36 - 4.62]
Black cohort	PH vs. PF mortality	1.35 [1.14 - 1.59]
White cohort	PH vs. PF mortality	0.45 [0.38 - 0.53]

When stratified by sex, around 40% of women and men who died had PF as the cause of death, followed by PH accounting for 33% of deaths in women and 22.5% in men. In a further subgroup analysis by race and gender, Black women with comorbid sarcoidosis, who are at the highest risk, were more likely to die from PH, accounting for approximately 40.5%. In contrast, among Black men, both PH and PF were equally responsible for two-thirds of total deaths (Figure 3). Interestingly, around 15% of Black men with comorbid sarcoidosis died from “other diseases of the respiratory system,” including respiratory failure and diseases not classified elsewhere. A subgroup analysis by race and gender was also performed for the White cohort; a consistently high burden of mortality due to PF was seen in both men and women, at 23.1% and 20.4%, respectively. Furthermore, PH was a less frequent cause of death in both men and women, at 6.6% and 10.3%, respectively.

## Discussion

Our analysis brings to light a distinct pattern in cause-specific pulmonary mortality among patients with comorbid sarcoidosis. Rather than focusing on trends already described in the existing literature [6], we highlight variation in causes of death by gender and race. PH emerged as the leading cause of pulmonary death in Black individuals, particularly Black women. This aligns with prior studies showing an overrepresentation of advanced pulmonary sarcoidosis and vascular involvement in this population [7]. PH is associated with a worse prognosis [8] and is also the leading cause of mortality in Black patients [7]. Our analysis further highlights this disparity, with Black patients facing a PH-related mortality 12 times more frequently than White patients (RR: 11.92; 95% CI: 9.96-14.28). Even within the Black cohort, the risk of death from PH was significantly higher than the risk of death from PF (RR: 1.35; 95% CI: 1.14-1.59). An analysis of the United Network for Organ Sharing (UNOS) database confirmed that higher pulmonary artery pressure is associated with lower survival among patients awaiting lung transplants [9].

The mechanisms of PH in sarcoidosis are multifactorial, ranging from vascular granulomas and pulmonary vasculopathy to chronic hypoxemia, left heart dysfunction, and even chronic thromboembolic states [10]. Sleep-disordered breathing, a known risk factor for PH, has also been observed at higher prevalence in sarcoidosis and may compound this risk in vulnerable populations [11]. Importantly, PH can occur independently of advanced fibrosis, and in some cases, even with near-normal pulmonary function [10]. This may explain why PH is the dominant cause of death in Black patients, even though they often show more radiographic fibrosis at diagnosis. In contrast to the PH-dominant mortality in Black patients, parenchymal destruction was the primary driver of mortality among White decedents. PF was more commonly listed as the cause of death in White patients, especially among males aged 55-64 years. Our calculation of RR within the White population showed that White patients were twice as likely to die from PF than from PH (RR for PH vs. PF: 0.45; 95% CI: 0.38-0.53). While Black patients maintained a higher risk of dying from PF compared with White patients (RR: 3.94; 95% CI: 3.36-4.62), the direction of PH versus PF disparity was opposite in the White population.

This suggests a different disease phenotype, one that progresses to fibrotic remodeling of the lung parenchyma. However, this presents a clinical paradox: prior studies have shown that Black patients with sarcoidosis often demonstrate more severe radiographic abnormalities [12], including fibrosis, compared with White patients. One possible explanation for fibrosis being listed more frequently in White patients is disease duration and survival bias. In our data and in other studies, White patients with sarcoidosis survived longer. This may allow fibrosis to progress further, eventually becoming the dominant and fatal feature. Swigris et al. [13] also supported the hypothesis that age is an independent risk factor for fibrosis. In contrast, earlier mortality in Black patients may limit fibrosis from becoming the documented cause of death, despite its radiographic presence. This survival bias hypothesis warrants validation in prospective cohort studies using longitudinal imaging and mortality data to determine whether the phenotypic differences we observed are driven by biologic differences or are an outcome of increased survival. These racial patterns become even more distinct when accounting for sex based differences in mortality.

This is most evident among Black women, in whom PH was disproportionately responsible for pulmonary deaths, echoing past studies showing that this group experiences higher disease severity and worse outcomes despite a younger age at diagnosis. Hormonal influences, barriers to healthcare access, and potential genetic susceptibility may all play roles in this trend. Conversely, fibrosis-related deaths were more common in men, especially White men, suggesting later-stage pulmonary involvement, possibly underdiagnosed or mischaracterized earlier in the disease course. These observed disparities, particularly the high mortality from PH in Black women (40.5%), suggest a distinct and severe clinical course compared with other cohorts.

This divergence stems from the distinct yet overlapping pathologies of sarcoidosis. While fibrosis is characterized by chronic parenchymal inflammation and scarring, contributing to mortality, especially in White patients, as our data reflects, PH, in comparison, can be secondary to fibrosis but can also arise from granulomatous vasculitis, pulmonary artery involvement, or left ventricular dysfunction in cardiac sarcoidosis. This clinical overlap is well documented in a study by Nunes, where 68.2% of patients with sarcoidosis and PH had radiographic stage IV disease. However, the authors also noted that around 53% of patients had PH that did not align with the severity of lung destruction [10]. Hence, the prevalence of PH-related deaths in Black patients may point toward increased vascular remodeling leading to PH and cardiac complications, possibly independent of extensive fibrosis.

Beyond PH and PF, infectious complications represent a significant and preventable cause of mortality in sarcoidosis. The third leading cause of pulmonary mortality in most patient groups with comorbid sarcoidosis was influenza and pneumonia. In a study conducted in Sweden, Larsson et al. reported an increased incidence of pneumonia and influenza among patients with sarcoidosis compared with controls [14]. Pneumococcal pneumonia is the most common bacterial cause of community-acquired pneumonia in adults [15]. A previous study from the Rochester Epidemiology Project compared the risk of hospital-acquired infections between patients with sarcoidosis and controls [16]. The HR was reported as 1.73 in untreated sarcoidosis patients, 3.3 in patients receiving low-dose glucocorticoids, and 4.48 in patients receiving glucocorticoids at doses greater than 10 mg per day. To address the increased risk of infections, the World Association of Sarcoidosis and Other Granulomatous Disorders has recommended the administration of inactivated vaccines, including pneumococcal, influenza, and hepatitis B vaccines, to all patients [17].

Overall, these findings support the growing call for cause-specific surveillance and management strategies in sarcoidosis, rather than a one-size-fits-all approach. Current guidelines recommend that patients with one or more risk factors for sarcoidosis-associated PH undergo screening with transthoracic echocardiography [18]. Based on the distinct mortality patterns observed in our study, we suggest the following clinical considerations: Routine PH screening should be prioritized in Black patients, particularly Black women with sarcoidosis, even if asymptomatic, due to their high mortality from PH [19]. In contrast, fibrosis-dominant sarcoidosis, as observed in our White cohort, may benefit from early antifibrotic strategies, as fibrosis appears to be the primary driver of mortality [20]. While these suggestions are based on population-level mortality trends, they offer a framework for risk-stratified management. The real-world feasibility and efficacy of these recommendations require further validation in future prospective clinical trials.

Limitations

This study has several important limitations. Firstly, the analysis is based on death certificate data, which may be subject to misclassification or underreporting. Furthermore, patients may have multiple pulmonary complications, such as both PF and PH, but only the most clinically apparent condition may be recorded, leading to underrepresentation of coexisting conditions. We also acknowledge that sarcoidosis itself can be the underlying cause in some cases. However, our focus was on pulmonary-specific complications in the context of sarcoidosis, and therefore, our methodology allowed us to analyze mortality from respiratory phenotypes in sarcoidosis rather than systemic sarcoidosis. In addition, as this is an ecological population-level study using aggregated national data, we were unable to evaluate individual-level clinical variables such as treatment exposure, disease duration, or imaging and functional parameters. Additionally, the use of ICD 10 codes relies on accurate documentation and coding practices that may vary over time or across geographic and racial groups.

Furthermore, structural inequities in healthcare access and socioeconomic status may impact the timing of diagnosis and the quality of care received across racial groups. Such disparities may also influence disease progression, especially the development of end-stage right heart failure or fibrotic complications, thereby affecting the reporting on death certificates. For instance, different patterns of clinical surveillance across hospital settings and population groups could introduce systematic bias, leading to certain complications being considered the primary cause of death and thereby contributing to observed phenotypic variations. While the CDC WONDER database provides a large sample size, it does not allow for the establishment of causality or adequate adjustment for potential confounders. Lastly, race and ethnicity are self-reported or family-reported and may not fully capture complex sociobiological interactions. Despite these limitations, our findings offer valuable insights into long term mortality trends and disparities and underscore the need for prospective studies.

## Conclusions

From 1999 to 2023, mortality with comorbid sarcoidosis showed an upward trend, with a major burden attributable to pulmonary complications. This analysis of CDC WONDER data also reveals racial disparity both quantitatively and qualitatively. Our analysis shows that White individuals tend to die from PF, whereas Black individuals more commonly die from pulmonary hypertension, highlighting a difference in clinical progression. While our findings are derived from population-level data and are subject to the coding limitations mentioned above, they highlight critical disparities that warrant further investigation. Future trials and cohort studies could examine the clinical progression of sarcoidosis across different populations. If the above relationship holds true, clinicians can maintain a high index of suspicion for PH in Black patients, while White patients may benefit from proactive evaluation and treatment of fibrotic lung disease. Ultimately, the effective management of sarcoidosis will require a multi-pronged approach that ensures early detection, individualized therapy, and structural health equity reforms. These reforms could include improved access to pulmonary and cardiology care, standardized screening guidelines for high-risk groups, and targeted education for high-risk populations.

## Appendices

**Table 3:** ICD-10 codes used to define respiratory diseases, pulmonary hypertension, and sarcoidosis for underlying and contributing causes of death ICD-10: the International Classification of Diseases, Tenth Revision

Diseases	ICD-10 code
Underlying and contributing causes of death	
Respiratory diseases	J00-J99: Diseases of the respiratory system
Pulmonary hypertension	I27.0-I27.89: Other pulmonary heart diseases
Sarcoidosis	D86: Sarcoidosis
Respiratory diseases	
Acute upper respiratory infections	J00-J06: Acute upper respiratory infections
Influenza and pneumonia	J09-J18: Influenza and Pneumonia
Acute bronchitis	J20-J22: Other acute lower respiratory infections
Rhinitis, pharyngitis, sinusitis	J30-J39: Other diseases of the upper respiratory tract
Chronic obstructive pulmonary disease	J40-J4A: Chronic lower respiratory diseases
Pneumoconiosis	J60-J70: Lung diseases due to external agents
Cardiogenic and non-cardiogenic pulmonary edema	J80-J84: Other respiratory diseases principally affecting the interstitium
Abscess and pyothorax	J85-J86: Suppurative and necrotic conditions of the lower respiratory tract
Pleural effusion and related diseases	J90-J94: Other diseases of the pleura
Iatrogenic pulmonary diseases	J95-J95: Intraoperative and postprocedural complications and disorders of the respiratory system, not elsewhere classified
Other diseases of the respiratory system	J96-J99: Other diseases of the respiratory system
